# Bromide promoted hydrogenation of CO_2_ to higher alcohols using Ru–Co homogeneous catalyst†
†Electronic supplementary information (ESI) available: GC-MS and GC data. See DOI: 10.1039/c6sc01314g


**DOI:** 10.1039/c6sc01314g

**Published:** 2016-04-18

**Authors:** Meng Cui, Qingli Qian, Zhenhong He, Zhaofu Zhang, Jun Ma, Tianbin Wu, Guanying Yang, Buxing Han

**Affiliations:** a Beijing National Laboratory for Molecular Sciences , CAS Key Laboratory of Colloid, Interface and Chemical Thermodynamics , Institute of Chemistry , Chinese Academy of Sciences , Beijing 100190 , China . Email: qianql@iccas.ac.cn ; Email: hanbx@iccas.ac.cn ; Tel: +86-10-62562821; b University of Chinese Academy of Sciences , Beijing 100049 , China

## Abstract

Iodides are commonly used promoters in C_2+_OH synthesis from CO_2_/CO hydrogenation. Here we report the highly efficient synthesis of C_2+_OH from CO_2_ hydrogenation over a Ru_3_(CO)_12_–Co_4_(CO)_12_ bimetallic catalyst with bis(triphenylphosphoranylidene)ammonium chloride (PPNCl) as the cocatalyst and LiBr as the promoter. Methanol, ethanol, propanol and isobutanol were formed at milder conditions. The catalytic system had a much better overall performance than those of reported iodide promoted systems because PPNCl and LiBr cooperated very well in accelerating the reaction. LiBr enhanced the activity and PPNCl improved the selectivity, and thus both the activity and selectivity were very high when both of them were used simultaneously. In addition, the catalyst could be reused for at least five cycles without an obvious change of catalytic performance.

## Abstract 2

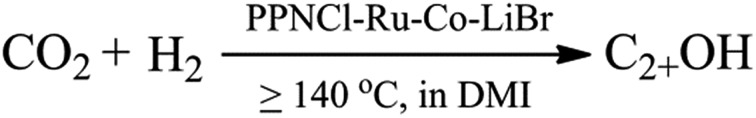
Higher alcohols can be synthesized efficiently by CO_2_ hydrogenation over Ru_3_(CO)_12_–Co_4_(CO)_12_ bimetallic catalyst with bis(triphenylphosphoranylidene)ammonium chloride (PPNCl) as the cocatalyst and LiBr as the promoter.

## Introduction

Carbon dioxide (CO_2_) is a greenhouse gas. On the other hand, it is an abundant, nontoxic, easily available, and renewable C1 resource.^1^ Transformation of CO_2_ into value-added chemicals is of great importance for the sustainable development of our society. Currently, utilization of CO_2_ as a feedstock to synthesize various chemicals,^2^ such as cyclic carbonates, carboxylic acids, methanol, formic acid, methyl formate and dimethylformamide, is investigated extensively.

Alcohols are important bulk chemicals. The synthesis of alcohols from CO_2_ hydrogenation has received much attention, but research progress was mainly focused on the synthesis of methanol.^3^ Higher alcohols (C_2+_OH) are more desirable in many cases, especially as fuel and fuel additives. However, the synthesis of C_2+_OH *via* CO_2_ hydrogenation is obviously a challenge. The acquired results for this topic are mostly focused on heterogeneous catalysis. For example, a CoMoS based catalyst produced 35.6% of C_2+_OH in alcohol products at 340 °C.^4^ An alkali-promoted Mo/SiO_2_ catalyst could generate alcohols at 250 °C with a C_2+_OH selectivity of 75.6%.^5^ A [Rh_10_Se]/TiO_2_ catalyst could catalyze the reaction at 350 °C with ethanol selectivity of 83%.^6^ A combined Rh–Fe–Cu based catalyst could produce ethanol with ethanol selectivity of about 70%.^7^ A C_2+_OH selectivity of 87.1% could be reached when modified K/Cu–Zn–Fe catalysts were used at 300 °C.^8^ It was found that water could promote C_2+_OH generation when a Pt/Co_3_O_4_ catalyst was used in a mixed solvent of water and DMI.^9^ In general, the activity and selectivity of C_2+_OH over heterogeneous catalysts were low and harsh reaction conditions were required.

Homogeneous catalysis is known for its higher catalytic efficiency compared to heterogeneous catalysis. But it has rarely been reported in CO_2_ hydrogenation to C_2+_OH, because the metal complexes are usually unstable in the reaction conditions. In the limited cases of homogeneous catalysis, iodides were used as a promoter and played a key role in the formation of C_2+_OH.^10^ But the catalytic systems suffer from low selectivity and/or low activity of C_2+_OH formation. For example, in the Ru–Co–KI system, only methanol and ethanol were generated and the ethanol selectivity was low (26.4%).10*a* When noble Rh was used to replace Co, the selectivity of C_2+_OH was improved but the activity was still low.10*b* Moreover, it is well known that iodides are the most commonly used promoters in C_2+_OH synthesis from CO_2_/CO hydrogenation because of their stronger nucleophilicity, which is favourable for generating larger alcohols.^10^–^12^ Bromides are much more stable and cheaper than iodides, but poor performances for generating higher alcohols limit their application in the reaction. Obviously, exploring more efficient and cheaper catalytic systems for the reaction is an interesting topic.

**Scheme 1:**
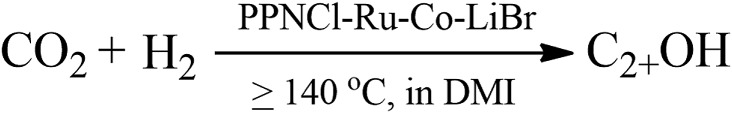
Synthesis of C_2+_OH from CO_2_ hydrogenation.

Herein we report the highly efficient synthesis of C_2+_OH from CO_2_ hydrogenation promoted by bromide using a Ru–Co bimetallic catalyst with PPNCl as the cocatalyst (Scheme 1). Methanol, ethanol, propanol and isobutanol were generated at milder conditions. The catalytic system had both a high activity and selectivity of C_2+_OH compared to those of iodide promoted reactions. In addition, the catalytic system could be recycled and reused at least five times without an obvious change of catalytic performance. As far as we know, this is the first work to use PPNCl as a cocatalyst in C_2+_OH synthesis *via* CO_2_ hydrogenation and we found that LiBr is a better promoter than LiI because PPNCl and LiBr cooperate effectively in enhancing the activity and selectivity.

## Results and discussion

Different catalytic systems were tested in the CO_2_ hydrogenation, and the results are shown in Table 1. In this work, space time yield (STY, in C mmol L^–1^ h^–1^) is used to show the activity of the catalytic systems, which is one of the commonly used units, especially when multi-metals are utilized. Using LiBr as the promoter, the reaction could proceed efficiently over the Ru_3_(CO)_12_/Co_4_(CO)_12_ bimetallic catalyst with PPNCl as the cocatalyst in 1,3-dimethyl-2-imidazolidinone (DMI) solvent (entry 1). The alcohols in the reaction solution were methanol, ethanol, propanol and isobutanol, and other products were negligible (Fig. S1†). Only two homogeneous catalytic systems have been reported for this reaction.10a,b The products in this work were different from those of CO_2_ hydrogenation by the Ru–Co–KI system, in which ethanol was the only C_2+_OH product.10*a* In previous work,10*b* we found that Ru–Rh–LiI was a very effective catalyst for producing C_2+_OH (12.9 C mmol L^–1^ h^–1^). Interestingly, the Ru–Co–PPNCl–LiBr catalyst designed in this work had much higher activity (33.7 C mmol L^–1^ h^–1^) with high selectivity, although cheap Co was used to replace Rh, indicating that LiBr and PPNCl played an important role for the very high activity and selectivity of the reaction, which will be discussed further in the following sections.

**Table 1:** Hydrogenation of CO_2_ to C_2+_OH using different catalytic systems^*a*^

Entry	Catalyst precursors	Promoter	Cocatalyst	Solvent	STY^*b*^ [C mmol L^–1^ h^–1^]					C_2+_OH Sel. [%]
					Methanol	Ethanol	Propanol	Isobutanol	Total	
1	Ru_3_(CO)_12_, Co_4_(CO)_12_	LiBr	PPNCl	DMI	3.1	29.5	0.6	0.5	33.7	90.8
2	Ru_3_(CO)_12_, Co_4_(CO)_12_	—	PPNCl	DMI	10.2	0.7	0.1	0	11.0	7.3
3	Ru_3_(CO)_12_, Co_4_(CO)_12_	LiBr	—	DMI	9.5	19.2	0.4	0.3	29.4	67.7
4^*c*^	Ru_3_(CO)_12_, Co_4_(CO)_12_	—	—	DMI	0.5	0	0	0	0.5	0
5	Ru_3_(CO)_12_, Co_4_(CO)_12_	LiCl	PPNCl	DMI	35.5	13.1	1.0	0	49.6	28.4
6	Ru_3_(CO)_12_, Co_4_(CO)_12_	LiI	PPNCl	DMI	0.4	1.9	3.9	0	6.2	93.5
7^*c*^	Ru_3_(CO)_12_, Co_4_(CO)_12_	LiBF_4_	PPNCl	DMI	2.7	0.3	0	0	3.0	10.0
8	Ru_3_(CO)_12_, Co_4_(CO)_12_	NaBr	PPNCl	DMI	42.4	2.5	0	0	44.9	5.6
9	Ru_3_(CO)_12_, Co_4_(CO)_12_	KBr	PPNCl	DMI	47.5	2.6	0	0	50.1	5.2
10	Ru_3_(CO)_12_, Co_4_(CO)_12_	KI	PPNCl	DMI	44.4	4.5	0	0	48.9	9.2
11	Ru_3_(CO)_12_	LiBr	PPNCl	DMI	12.1	20.8	0	0	32.9	63.2
12	Co_4_(CO)_12_	LiBr	PPNCl	DMI	0.3	0	0	0	0.3	0
13	Ru_3_(CO)_12_, Co_4_(CO)_12_	LiBr	LiCl	DMI	1.3	5.9	0.5	0.2	7.9	83.5
14	Ru_3_(CO)_12_, Co_4_(CO)_12_	LiBr	TBACl	DMI	8.9	23.4	0.8	0.7	33.8	73.7
15	Ru_3_(CO)_12_, Co_4_(CO)_12_	LiBr	TPPTS	DMI	10.1	13.3	0.2	0	23.6	57.2
16	Ru_3_(CO)_12_, Co_4_(CO)_12_	LiBr	PPh_3_	DMI	10.9	13.7	0.3	0	24.9	56.2
17	Ru_3_(CO)_12_, Co_4_(CO)_12_	LiBr	Imidazole	DMI	5.1	11.5	0	0	16.6	69.3
18	Ru_3_(CO)_12_, Co_4_(CO)_12_	LiBr	PPNCl	NMP	8.7	13.6	4.7	4.0	31.0	71.9
19	Ru_3_(CO)_12_, Co_4_(CO)_12_	LiBr	PPNCl	DMF	8.2	0	0	0	8.2	0
20	Ru_3_(CO)_12_, Co_4_(CO)_12_	LiBr	PPNCl	[Bmim]NTf_2_	1.1	0	0	0	1.1	0
21	Ru_3_(CO)_12_, Co_4_(CO)_12_	LiBr	PPNCl	1-Methylpiperidine	0	0	0	0	0	0
22	Ru_3_(CO)_12_, Co_4_(CO)_12_	LiBr	PPNCl	THF	71.4	2.3	0	0	73.7	3.1
23	Ru_3_(CO)_12_, Co_4_(CO)_12_	LiBr	PPNCl	Cyclohexane	1.2	0.1	0	0	1.3	7.7
24^*c*^	Ru_3_(CO)_12_, Co_4_(CO)_12_	LiBr	PPNCl	H_2_O	2.1	1.1	0.1	0	3.3	36.4
25	Ru_3_(CO)_12_, Co_2_(CO)_8_	LiBr	PPNCl	DMI	10.0	22.6	1.1	0	33.7	70.3
26^*c*^	RuBr_3_, CoBr_2_	LiBr	PPNCl	DMI	4.2	8.5	0	0	12.7	66.9
27	(PPh_3_)_3_RuCl_2_, (PPh_3_)_3_CoCl	LiBr	PPNCl	DMI	3.1	4.2	0	0	7.3	57.5

^*a*^Reaction conditions: 40 μmol Ru catalyst and 20 μmol Co catalyst (based on the metal), 4 mmol promoter, 0.15 mmol cocatalyst, 2 mL solvent, 3 MPa CO_2_ and 6 MPa H_2_ (at room temperature), 200 °C, 12 h.

^*b*^STY stands for space time yield (C mmol L^–1^ h^–1^). The STY was determined by GC analysis using toluene as the internal standard.

^*c*^Black precipitate was observed after the reaction. Sel.: selectivity.

The LiBr promoter played a crucial role in accelerating the reaction. Without LiBr, both the activity and selectivity of the C_2+_OH synthesis were very low (entry 2). The LiBr also enhanced the stability of the catalyst. When LiBr was used without PPNCl, the catalyst was also very active, but the selectivity to C_2+_OH was much lower (entry 3). This indicates that LiBr and PPNCl cooperated very well for producing C_2+_OH. LiBr enhanced the activity and PPNCl improved the selectivity. Thus, both the activity and selectivity were very high when both of them were present. A black metal precipitate was observed if both LiBr and PPNCl were absent (entry 4). We also tested the promoters with other cations (Na^+^ and K^+^) and anions (Cl^–^, I^–^ and BF_4_^–^), but the activity and/or selectivity of the catalyst were poor (entries 5–10). The contribution of the lithium halide to C_2+_OH selectivity followed the order of: LiI > LiBr > LiCl, while their contribution to the activity (STY) follows the reverse order (entries 1, 5 and 6). The data show that the selectivities of the catalytic systems with LiBr and LiI were similar, but the activity of the catalytic system with LiBr was much higher (entries 1 and 6). The excellent performance of the catalytic system with LiBr can be attributed mainly to the presence of PPNCl, which enhanced the selectivity significantly, whilst retaining the high activity (entries 1 and 3). Thus LiBr was the best promoter for the above Ru–Co–PPNCl catalyst in this reaction. In the previous work, the single Ru catalyst using an iodide promoter had very poor performance for producing C_2+_OH.^10^ While in this work, the ethanol selectivity could reach 63.2% when the Ru–PPNCl catalyst was promoted by LiBr (entry 11). The Co catalyst itself showed very poor catalytic performance (entry 12). But, when it was combined with Ru catalyst, propanol and isobutanol were produced and the selectivity of C_2+_OH increased to 90.8% (entry 1). Hence a synergistic effect existed in the Ru–Co–PPNCl catalysts. The PPNCl was important for the catalytic properties. Without PPNCl, the catalytic performance, especially the C_2+_OH selectivity, was much lower (entry 3). We also tried other cocatalysts, but the results were not satisfactory (entries 13–17). The X-ray photoelectron spectroscopy (XPS) study revealed the coordination between Ru_3_(CO)_12_ and Cl^–^ in PPNCl (Fig. S2†). The coordination increased the electron density of the Ru atom and could promote the oxidative addition of alkyl halides to the active center, which is usually a key step in chain growth reactions.^12^ The superiority of PPNCl over other chlorides (entries 1, 13 and 14) may be due to the big steric hindrance of the substituents around the N atom, which weakened the electrostatic attraction between PPN^+^ and Cl^–^ and the nucleophilicity of the Cl^–^ was enhanced accordingly.^13^,^14^ After screening the solvents, we found that DMI was the best for the reaction (entries 18–24). We also tried other Ru–Co combinations, but the efficiency was lower than that of Ru_3_(CO)_12_ and Co_4_(CO)_12_ (entries 1 and 25–27). The Co_2_(CO)_8_ adopted in the literature10*a* was not suitable here (entry 25).

The impact of catalyst dosage and gas pressure on the reaction was studied and the results are given in Table 2. When the total dosage of Ru and Co catalysts was fixed, the optimized ratio of Ru/Co was 2 : 1 (entries 1–4 of Table 2). As expected, the increase of the catalyst dosage enhanced the catalytic efficiency, but it was less sensitive when the dosage was large enough (entries 3, 5 and 6 of Table 2). At a fixed ratio of CO_2_ and H_2_, the STY of the C_2+_OH increased rapidly with elevating pressure (entries 3, 7 and 8 of Table 2). The optimal ratio of CO_2_ and H_2_ was 1 : 2 at a given total pressure (entries 3 and 9–11 of Table 2).

**Table 2:** Effect of reaction parameters on the synthesis of C_2+_ alcohols by CO_2_ hydrogenation^*a*^

Entry	Ru/Co [μmol]	*p*CO_2_ [MPa]	*p*H_2_ [MPa]	STY [C mmol L^–1^ h^–1^]					C_2+_OH Sel. [%]
				Methanol	Ethanol	Propanol	Isobutanol	Total	
1	20/40	3	6	7.5	18.8	0.2	0.1	26.6	71.8
2	30/30	3	6	7.3	22.3	0.5	0.3	30.4	76.0
3	40/20	3	6	3.1	29.5	0.6	0.5	33.7	90.8
4	45/15	3	6	9.5	23.5	0.6	0.4	34.0	72.1
5	20/10	3	6	9.9	17.9	0.4	0.2	28.4	65.1
6	60/30	3	6	2.9	30.5	0.8	0.7	34.9	91.7
7	40/20	1	2	0.4	0	0	0	0.4	0
8	40/20	2	4	1.6	12.8	0.6	0.6	15.6	89.7
9	40/20	2.25	6.75	13.2	25.9	0.7	0.4	40.2	67.2
10	40/20	4.5	4.5	3.4	17.8	1.0	0.4	22.6	85.0
11	40/20	6	3	1.7	6.1	0.3	0.2	8.3	79.5

^*a*^Reaction conditions: Ru_3_(CO)_12_ and Co_4_(CO)_12_ were used as catalyst precursors and their dosage was based on the metal, 4 mmol LiBr, 0.15 mmol PPNCl, 2 mL DMI, 200 °C, 12 h. Sel.: selectivity.

**Fig. 1:**
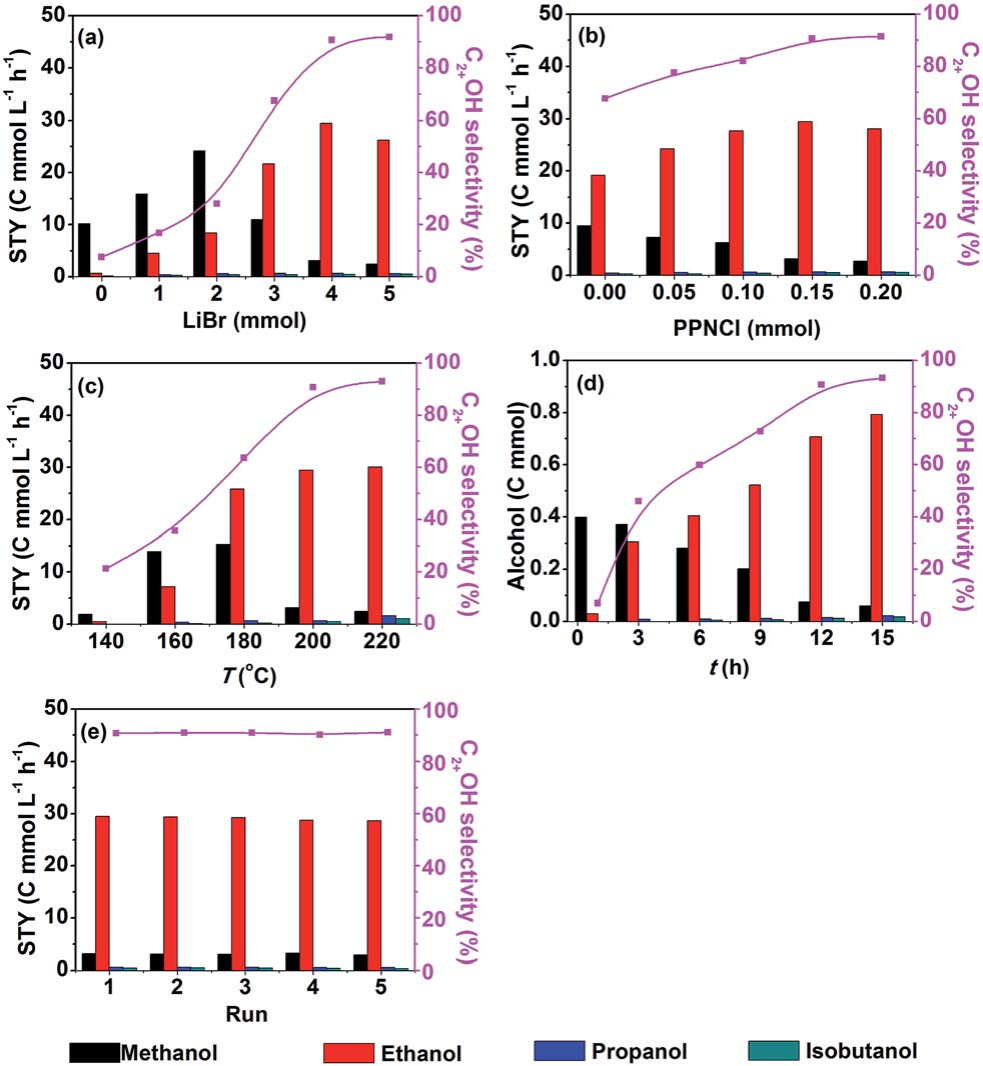
Effect of reaction conditions (a–d) and results of recycling tests (e) over 40 μmol Ru_3_(CO)_12_ and 20 μmol Co_4_(CO)_12_ (based on the metal) in DMI under 9 MPa of the initial pressure (CO_2_/H_2_ = 1/2): (a) effect of LiBr dosage, 0.15 mmol PPNCl, 200 °C, 12 h; (b) effect of PPNCl dosage, 4 mmol LiBr, 200 °C, 12 h; (c) effect of reaction temperature, 4 mmol LiBr, 0.15 mmol PPNCl, 12 h; (d) effect of reaction time, 4 mmol LiBr, 0.15 mmol PNNCl, 200 °C; (e) the reaction condition is the same as that of entry 1 in Table 1.

Fig. 1a shows that the STY and selectivity of C_2+_OH were enhanced significantly by increasing the LiBr dosage from 0–4 mmol. When LiBr usage was further increased the STY of C_2+_OH decreased notably. Thus, the suitable dosage of LiBr was 4 mmol. In contrast, the STY of methanol increased evidently when the LiBr usage increased from 0–2 mmol, whereas it decreased drastically with further increasing of the LiBr dosage. It is obvious that LiBr played a key role in generating methanol and transforming it into C_2+_OH. In addition, Br^–^ would occupy the active sites of the catalyst and inhibit the reaction when its dosage was high enough. The effect of PPNCl dosage on the reaction is depicted in Fig. 1b. With the increase of PPNCl dosage, the STY of C_2+_OH improved gradually, but it dropped when the dosage exceeded 0.15 mmol, which may be due to occupation of the active sites. In contrast, the STY of methanol always decreased with increasing the dosage of PPNCl. Hence the appropriate dosage of PPNCl was 0.15 mmol. The above results also support the conclusion that the PPNCl promotes the transformation of methanol into C_2+_OH.

The impact of reaction temperature is demonstrated in Fig. 1c. Methanol and ethanol began to emerge at 140 °C. A minor amount of propanol and isobutanol appeared at 160 °C. With the increase of reaction temperature, the STY and selectivity of C_2+_OH enhanced evidently, but it became insensitive when the temperature was above 200 °C. So a suitable reaction temperature was 200 °C. The time course of the reaction is shown in Fig. 1d. Methanol and ethanol were formed in 1 h. At 3 h and 6 h, propanol and isobutanol began to appear, respectively. With time going on, the yield and selectivity of C_2+_OH increased rapidly, while the methanol content kept decreasing. After 12 h, the change of methanol content was not evident and growth of the ethanol yield became slower, and at the same time, the yield of propanol and isobutanol increased. As a whole, the variation of C_2+_OH selectivity after 12 h was not obvious. Fig. 1d also demonstrates that the amount of C_2+_OH showed nearly a linear increase with reaction time, suggesting that the water generated *in situ* had no considerable influence on the activity of the catalyst.

We also studied the recyclability of the catalytic system. After the reaction, the alcohols generated in the reaction were removed under vacuum, which was confirmed by gas chromatography. Then the catalytic system was reused directly for the next run. The results of the recycling test are shown in Fig. 1e. The STY and selectivity of C_2+_OH did not decrease obviously after five cycles (12 h each cycle), indicating that the catalytic system had excellent stability and reusability.

As is shown in Fig. 1d, methanol is firstly generated and gradually consumed. At the same time, the C_2+_OH increased accordingly. These phenomena suggest that methanol was first formed from CO_2_ hydrogenation, and then acted as an intermediate to produce larger alcohols. To support this assumption, we conducted tracer experiments by adding a small amount of ^13^CH_3_OH into the reaction. The GC-MS data indicated that ^13^C appeared in all of the target C_2+_OH (Fig. S3†), supporting the above argument. We also tried ^13^C_2_H_5_OH and obtained a similar result (Fig. S4†). Thus it can be concluded that in CO_2_ hydrogenation to generate the alcohols, the small alcohols acted as building blocks for the larger ones.

**Scheme 2:**
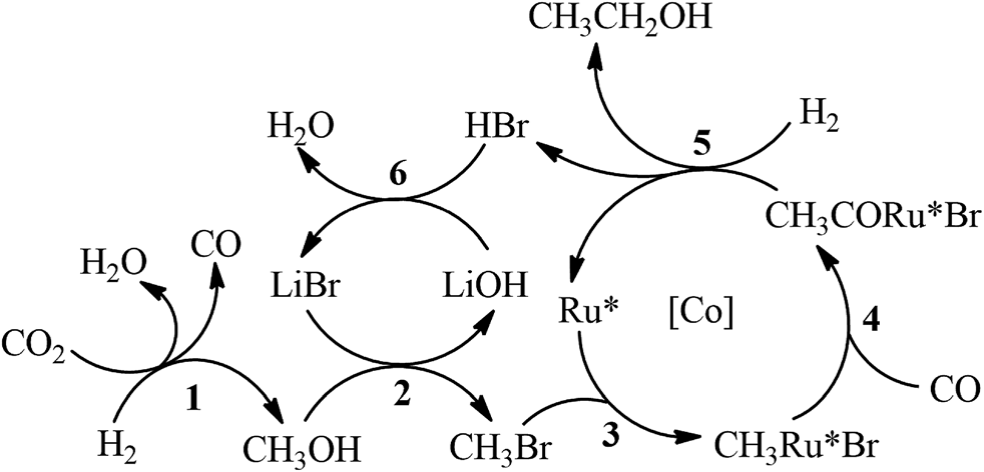
Proposed mechanism of C_2+_OH synthesis from CO_2_ hydrogenation.

The possible mechanism for the synthesis of C_2+_OH from CO_2_ hydrogenation is depicted in Scheme 2. Methanol and CO were generated *via* Ru catalyzed CO_2_ hydrogenation (Step 1). The methanol generated *in situ* was further converted into ethanol *via* a hydrocarbonylation reaction (Steps 2–5). The generation of propanol from ethanol should follow similar steps. Minor isobutanol was formed *via* the Guerbet reaction between methanol and propanol.^15^ The Ru–halide catalyzed synthesis of methanol and CO from CO_2_ hydrogenation has been reported elsewhere.^16^ The mechanism of methanol hydrocarboxylation using Ru–Co–iodide systems has been extensively investigated.^12^ The Co catalyst was mainly responsible for the generation of ethanol and acetaldehyde from methanol hydrocarbonylation, and the Ru catalyst further hydrogenated the acetaldehyde into ethanol. However, in this work, the bromide promoted Ru catalyst predominated the production of ethanol from methanol (entry 11 of Table 1), while the single Co complex could not effectively catalyze the reaction (entry 12 of Table 1). The Co catalyst ([Co]) in this work mainly accelerated the generation of C_2+_OH in the reaction. The coordination between the active Ru center (Ru*) and Cl^–^ from PPNCl enhanced the electron density of the metal center, which would expedite the oxidative addition step (Step 3).^17^ Meanwhile, the increase of the electron density on the Ru* could promote the hydrogenation step (Step 5).^12^

## Conclusions

In summary, we have investigated different catalytic systems for hydrogenation of CO_2_ to C_2+_OH. It was discovered that LiBr could promote the reaction very efficiently using a Ru–Co bimetallic catalyst with PPNCl as the cocatalyst. The catalytic system could be reused five times without an obvious loss of catalytic performance. A synergistic effect existed between the Ru and Co catalyst. Moreover, PPNCl enhanced the selectivity of the catalytic system with LiBr significantly, while keeping its very high activity. Therefore, both the selectivity and activity were very high in the presence of PPNCl and LiBr. The outstanding performance of the catalytic system results from the cooperative effect of Ru, Co, PPNCl, and LiBr. Very interestingly, the results of this work demonstrate that bromide is a much better promoter than iodide in C_2+_OH synthesis *via* CO_2_ hydrogenation because the bromide promoter worked cooperatively with PPNCl. We believe that other bromide promoted catalytic systems with excellent performance can be explored for the synthesis of alcohols from CO_2_ hydrogenation. It is also instructive for designing catalysts of CO hydrogenation.

## Experimental section

### Chemicals

Dodecacarbonyltriruthenium (Ru_3_(CO)_12_, 99%), dichlorotris(triphenylphosphine)ruthenium(ii) ((PPh_3_)_3_RuCl_2_, 97%), ruthenium(iii) bromide hydrate (RuBr_3_·*x*H_2_O, Ru 25% min), dodecacarbonyltetracobalt (Co_4_(CO)_12_, 98%), chlorotris(triphenyl phosphine)cobalt(i) ((PPh_3_)_3_CoCl, 97%), cobalt(ii) bromide (CoBr_2_, 97%), lithium bromide (LiBr, 99%), lithium iodide (LiI, 99.95%), lithium tetrafluoroborate (LiBF_4_, 98%), sodium bromide (NaBr, 99%), potassium bromide (KBr, 99%), potassium iodide (KI, 99.9%), bis(triphenylphosphoranylidene)ammonium chloride (PPNCl, 97%), triphenylphosphine (PPh_3_, 99%), imidazole (99%), 1-methyl-2-pyrrolidinone (NMP, 99%), *N*,*N*-dimethylformamide (DMF, 99%), 1-methylpiperidine (99%), tetrahydrofuran (THF, 99%), methanol, and cyclohexane (99%) were purchased from Alfa Aesar China Co, Ltd. Dicobalt octacarbonyl (Co_2_(CO)_8_), lithium chloride (LiCl, 98%), tetrabutylammonium chloride (TBACl, 98%), and 1,3-dimethyl-2-imidazolidinone (DMI, 98%) were provided by TCI Shanghai Co., Ltd. Tris(3-sulfonatophenyl)phosphine sodium salt hydrate (TPPTS, 95%) was obtained from J&K Scientific Ltd. 1-Butyl-3-methylimidazolium bis(trifluoromethylsulfonyl)imide ([Bmim]-NTf_2_, 99%) was purchased from Centre of Green Chemistry and Catalysis, LICP, CAS. Toluene (99.8%) was obtained from Xilong Chemical Co., Ltd. Methanol–^13^C (99 atom% ^13^C) and ethanol–^13^C_2_ (99 atom% ^13^C) were provided by Sigma-Aldrich Co. LLC. CO_2_ (99%) and H_2_ (99%) were supplied by Beijing Analytical Instrument Company. All chemicals were used as received.

### Hydrogenation of CO_2_

The reactions were carried out in a 16 mL Teflon-lined stainless steel reactor equipped with a magnetic stirrer. In a typical experiment, the desired amount of catalyst, cocatalyst, promoter, tracer (if used) and 2 mL solvent were added into the reactor. After the air in the reactor was removed under vacuum, CO_2_ and H_2_ were charged into the reactor to the desired pressure at room temperature. Then the reactor was placed in an air bath of constant temperature, and the stirrer was started at 800 rpm. After reaction, the reactor was cooled in an ice-water bath and the residual gas was released carefully in a hood. The reaction solution was analyzed by a gas chromatograph (GC, Agilent 7890B) equipped with a flame ionization detector (FID) and a HP-5 capillary column. Toluene was used as the internal standard. Identification of the liquid products was done using GC-MS (SHIMADZU-QP2010) as well as by comparing the retention times with respective standards in the GC traces.

To test the reusability of the catalytic system, the alcohols formed in the reaction were removed under vacuum at 80 °C for 2 h, and then the catalytic system was reused directly for the next run.

## Supplementary Material

Supplementary information
