# Supplementary material for: Bromide promoted hydrogenation of CO2 to higher alcohols using Ru–Co homogeneous catalyst
Source: Chem Sci. 2016 Apr 18;7(8):5200–5. doi: 10.1039/c6sc01314g (PMC6020613; doi:10.1039/c6sc01314g)
Supplement: Supplementary file 1 [file SC-007-C6SC01314G-s001.pdf]

Electronic Supplementary Material (ESI) for Chemical Science.
This journal is © The Royal Society of Chemistry 2016

Electronic Supplementary Information

Bromide promoted hydrogenation of CO_2 to higher alcohols using Ru-Co homogeneous catalyst

Meng Cui, Qingli Qian,* Zhenhong He, Zhaofu Zhang, Jun Ma, Tianbin Wu, Guanying Yang, Buxing Han*

Beijing National Laboratory for Molecular Sciences, CAS Key Laboratory of Colloid, Interface and Chemical Thermodynamics, Institute of Chemistry, Chinese Academy of Sciences, Beijing100190, China. E-mail: qianql@iccas.ac.cn; hanbx@iccas.ac.cn

Figures

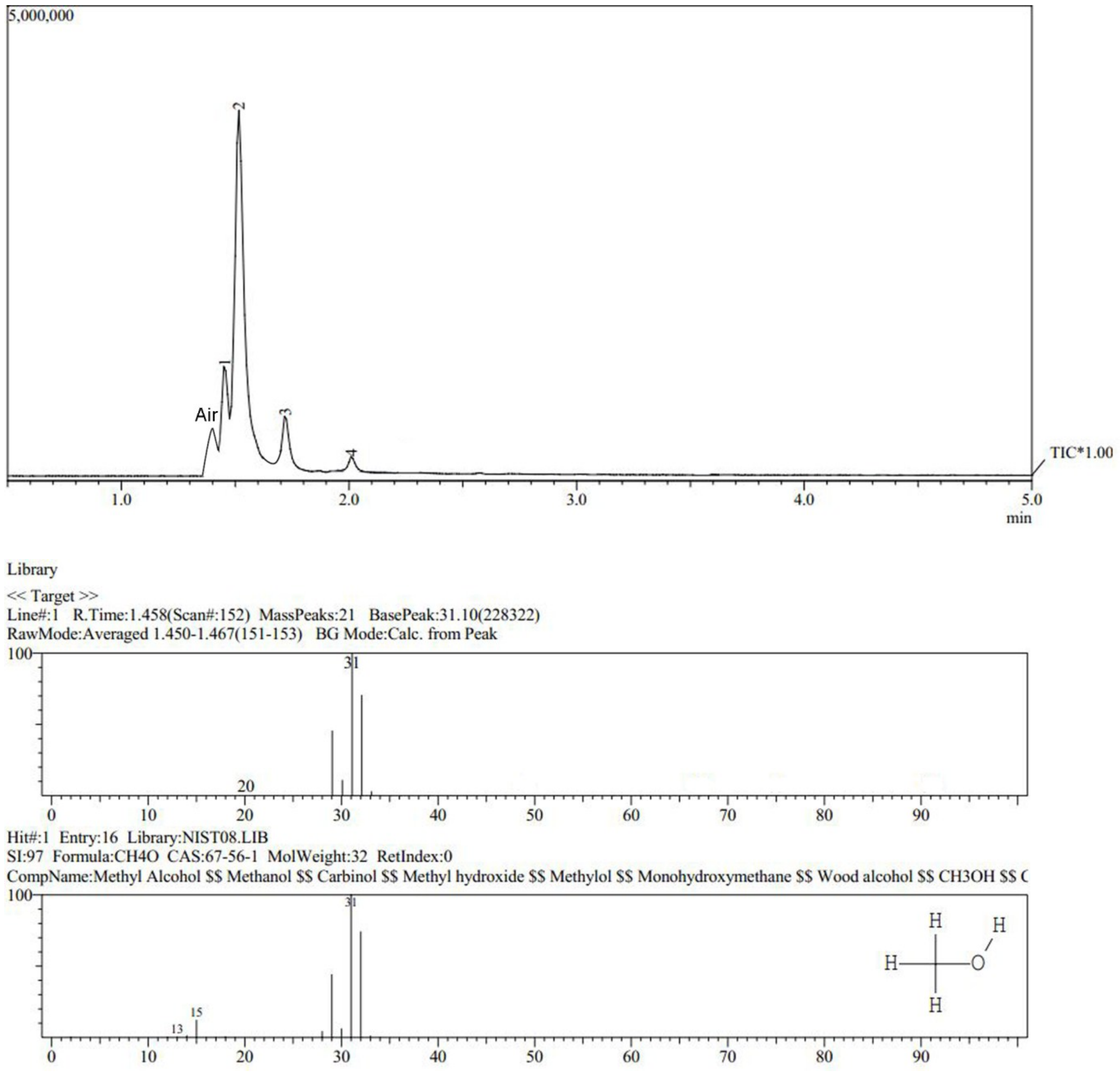

5,000,000
Air
TIC*1.00
1.0
2.0
3.0
4.0
5.0
min
Library
<< Target >>
Line#:1 R.Time:1.458(Scan#:152) MassPeaks:21 BasePeak:31.10(228322)
RawMode:Averaged 1.450-1.467(151-153) BG Mode:Calc. from Peak
Hit#:1 Entry:16 Library:NIST08.LIB
SI:97 Formula:CH4O CAS:67-56-1 MolWeight:32 RetIndex:0
CompName:Methyl Alcohol $$ Methanol $$ Carbinol $$ Methyl hydroxide $$ Methylol $$ Monohydroxymethane $$ Wood alcohol $$ CH3OH $$ C

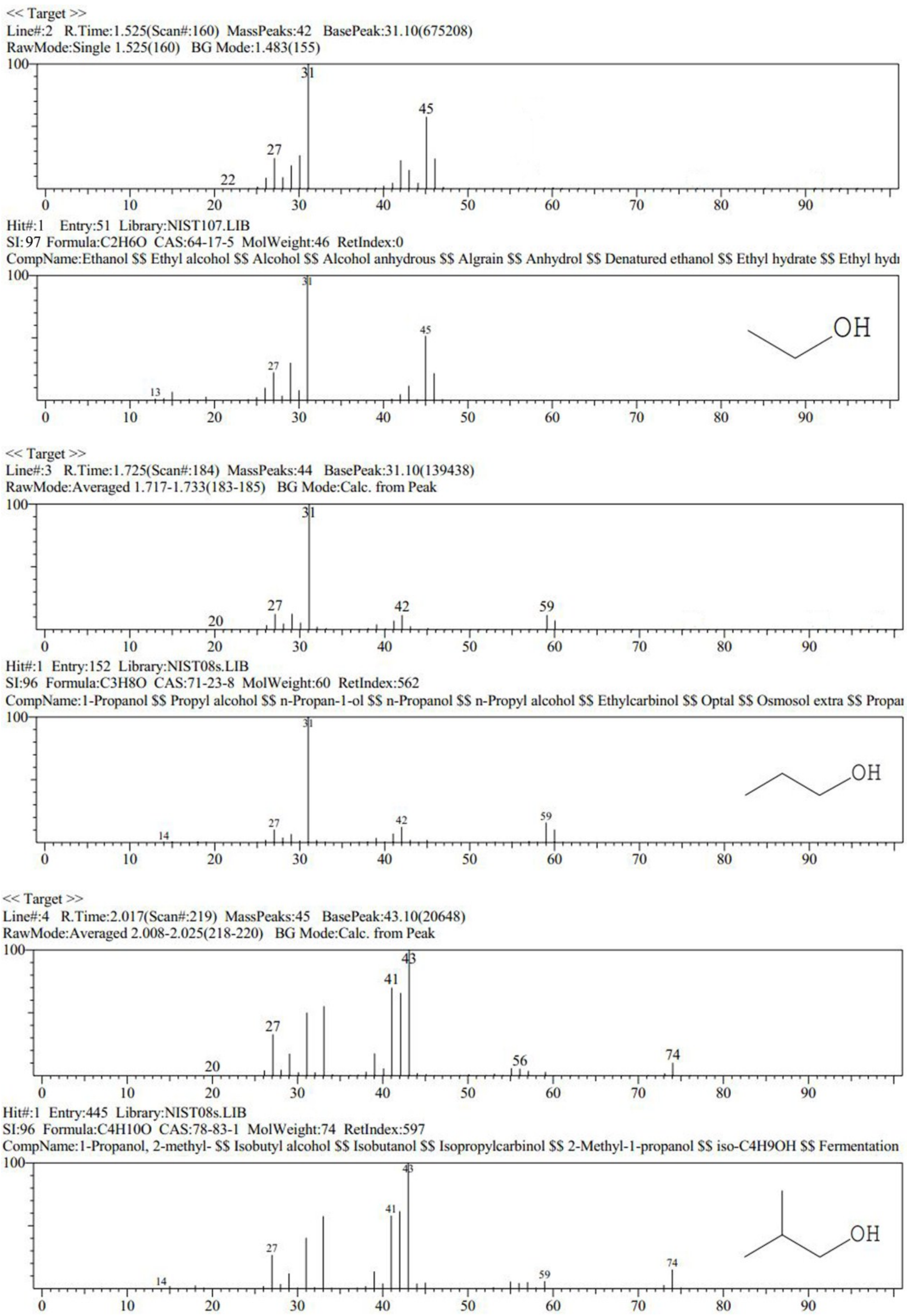

Fig. S1 GC-MS spectra of the products. Reaction conditions were the same as that of entry 1 in Table 1.

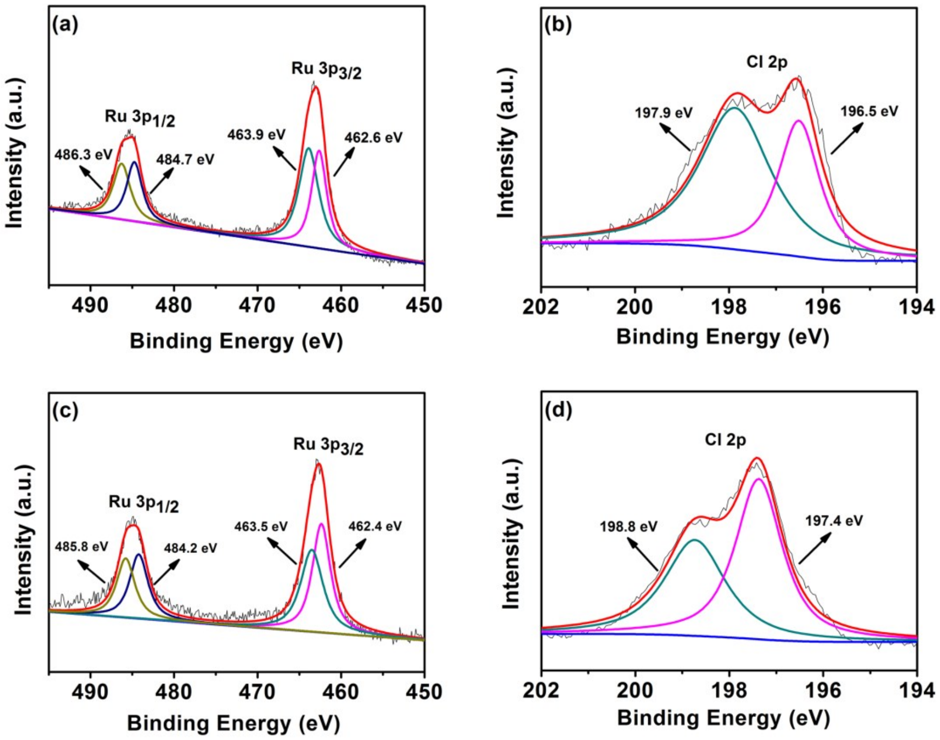

Fig. S2 XPS spectra of Ru (a), Cl (b) before the coordination of $Ru_3(CO)_{12}$ with PPNCl and Ru (c), Cl (d) after the coordination of $Ru_3(CO)_{12}$ with PPNCl.

Notes: The coordination of $Ru_3(CO)_{12}$ and PPNCl was conducted as follows: 8.5 mg $Ru_3(CO)_{12}$ (40 μmol Ru) was dissolved in 20 mL THF, 86.1 mg PPNCl (0.15 mmol) was dissolved in 20 mL methanol, the above solutions were mixed under stirring and the color of the mixed solution changed gradually from orange to bright yellow. After the mixture was stirred for 5 hrs at 40 ^{o}C, the solvent was removed using rotary evaporator. The precipitate was dried in vacuum at room temperature for 12 hrs before the XPS analysis. The X-ray photoelectron spectroscopy (XPS) data were obtained with an ESCA Lab 220i-XL electron spectrometer from VG Scientific using 300 W AlKα radiation. The base pressure was about 3×10^{-9} mbar. The binding energy was referenced to the C_{1s} line at 284.8 eV from adventitious carbon.

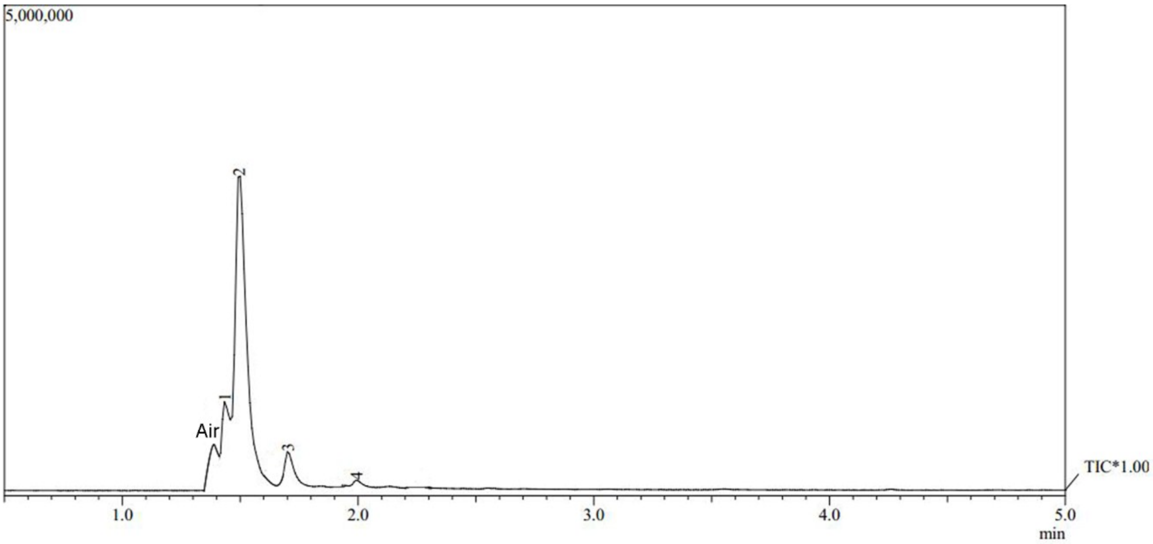

Library
<< Target >>
Line#:1 R.Time:1.433(Scan#:149) MassPeaks:21 BasePeak:31.10(167434)
RawMode:Averaged 1.425-1.442(148-150) BG Mode:Calc. from Peak

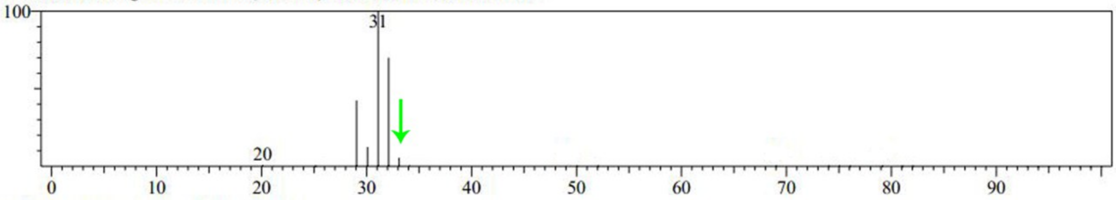

Hit#:1 Entry:15 Library:NIST147.LIB
SI:96 Formula:CH4O CAS:67-56-1 MolWeight:32 RetIndex:0
CompName:Methyl Alcohol $$ Methanol $$ Carbinol $$ Methyl hydroxide $$ Methylol $$ Monohydroxymethane $$ Wood alcohol $$ CH3OH $$ C

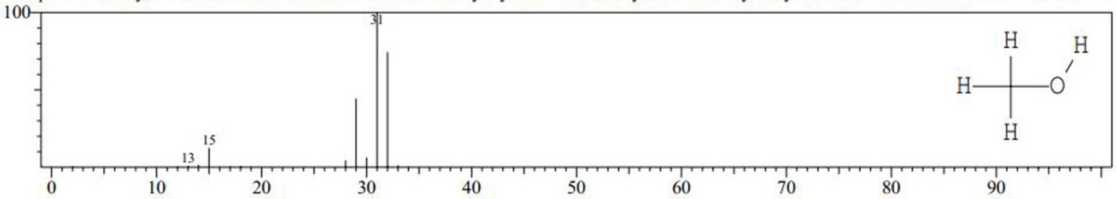

<< Target >>
Line#:2 R.Time:1.508(Scan#:158) MassPeaks:45 BasePeak:31.10(800308)
RawMode:Single 1.508(158) BG Mode:1.600(169)

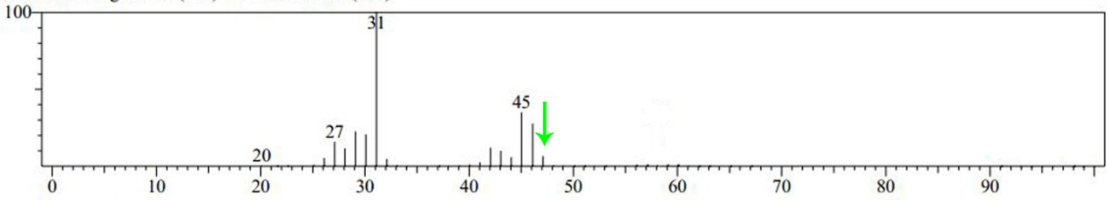

Hit#:1 Entry:60 Library:NIST21.LIB
SI:84 Formula:C2H6O CAS:64-17-5 MolWeight:46 RetIndex:0
CompName:Ethanol

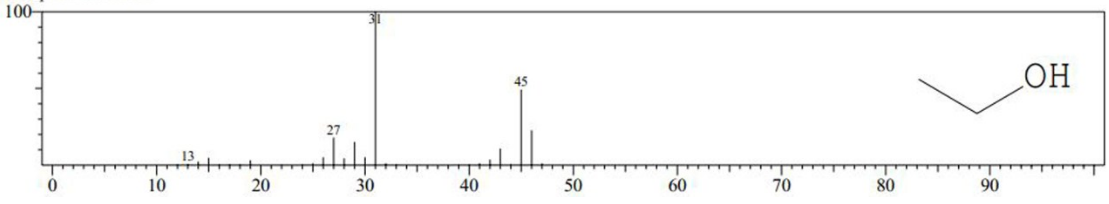

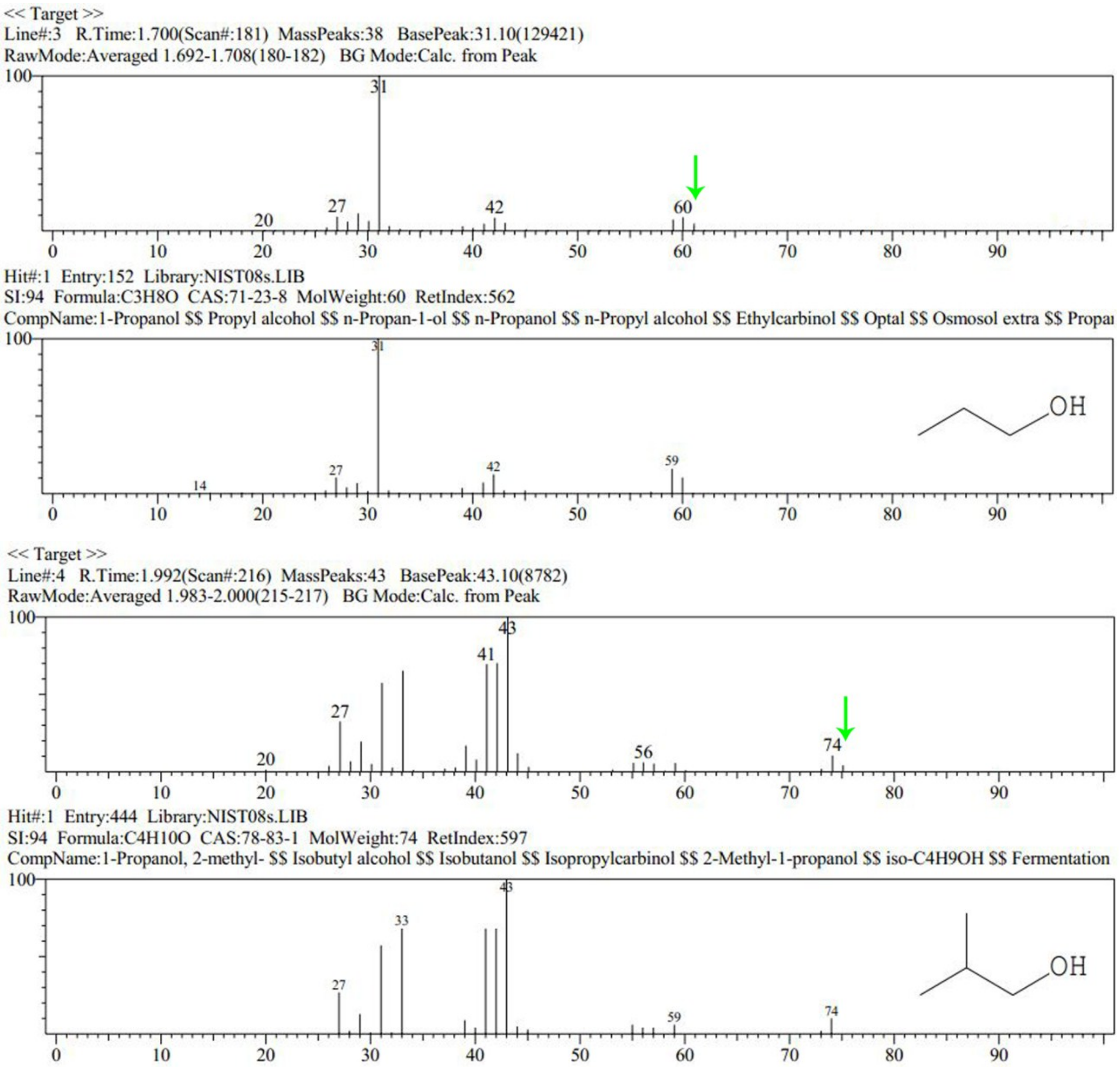

Fig. S3 GC-MS spectra of $^{13}CH_3OH$ tracer experiment. 60 μL $^{13}CH_3OH$ was used as tracer, other reaction conditions were the same as that of entry 1 in Table 1.

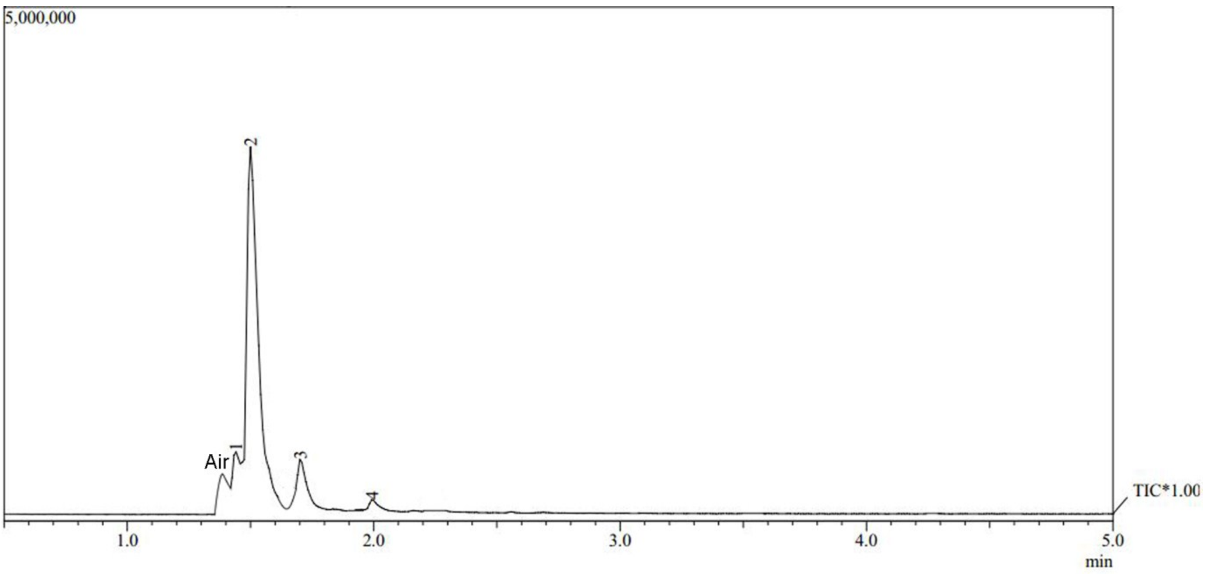

Library

<< Target >>

Line#:1 R.Time:1.442(Scan#:150) MassPeaks:23 BasePeak:31.10(130043)

RawMode:Averaged 1.433-1.450(149-151) BG Mode:Calc. from Peak

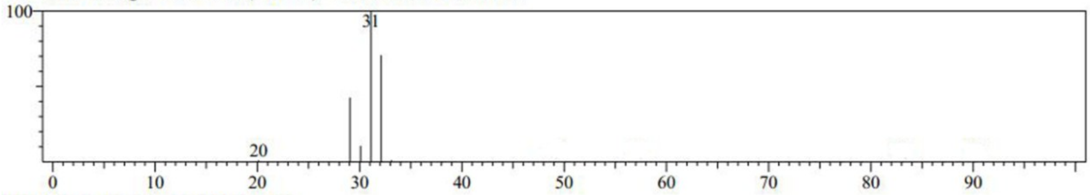

Hit#:1 Entry:15 Library:NIST147.LIB

SI:97 Formula:CH4O CAS:67-56-1 MolWeight:32 RetIndex:0

CompName:Methyl Alcohol $$ Methanol $$ Carbinol $$ Methyl hydroxide $$ Methylol $$ Monohydroxymethane $$ Wood alcohol $$ CH3OH $$ C

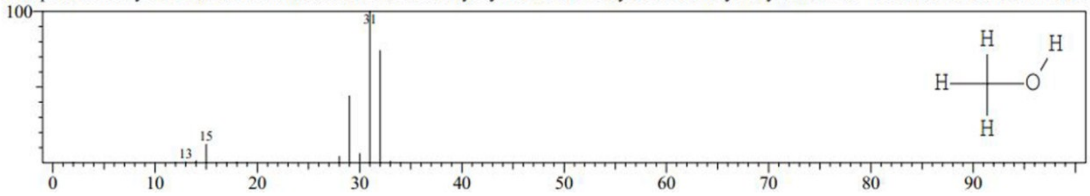

<< Target >>

Line#:2 R.Time:1.500(Scan#:157) MassPeaks:48 BasePeak:31.10(480053)

RawMode:Averaged 1.492-1.508(156-158) BG Mode:Calc. from Peak

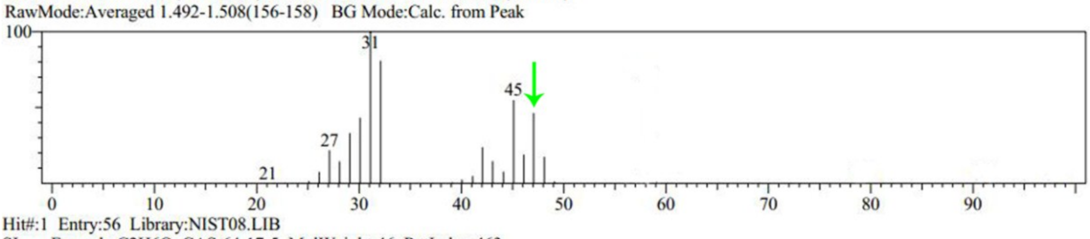

Hit#:1 Entry:56 Library:NIST08.LIB

SI: Formula:C2H6O CAS:64-17-5 MolWeight:46 RetIndex:463

CompName:Ethanol $$ Ethyl alcohol $$ Alcohol $$ Alcohol anhydrous $$ Algrain $$ Anhydrol $$ Denatured ethanol $$ Ethyl hydrate $$ Ethyl hydı

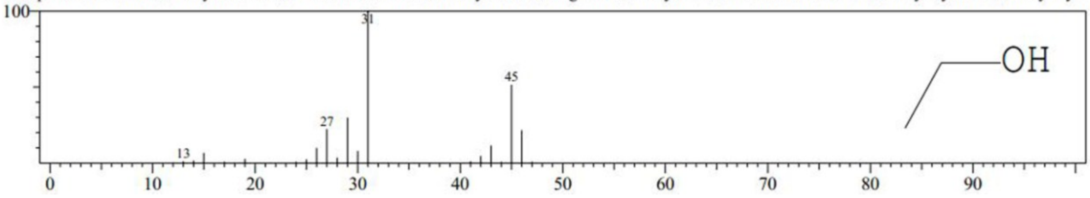

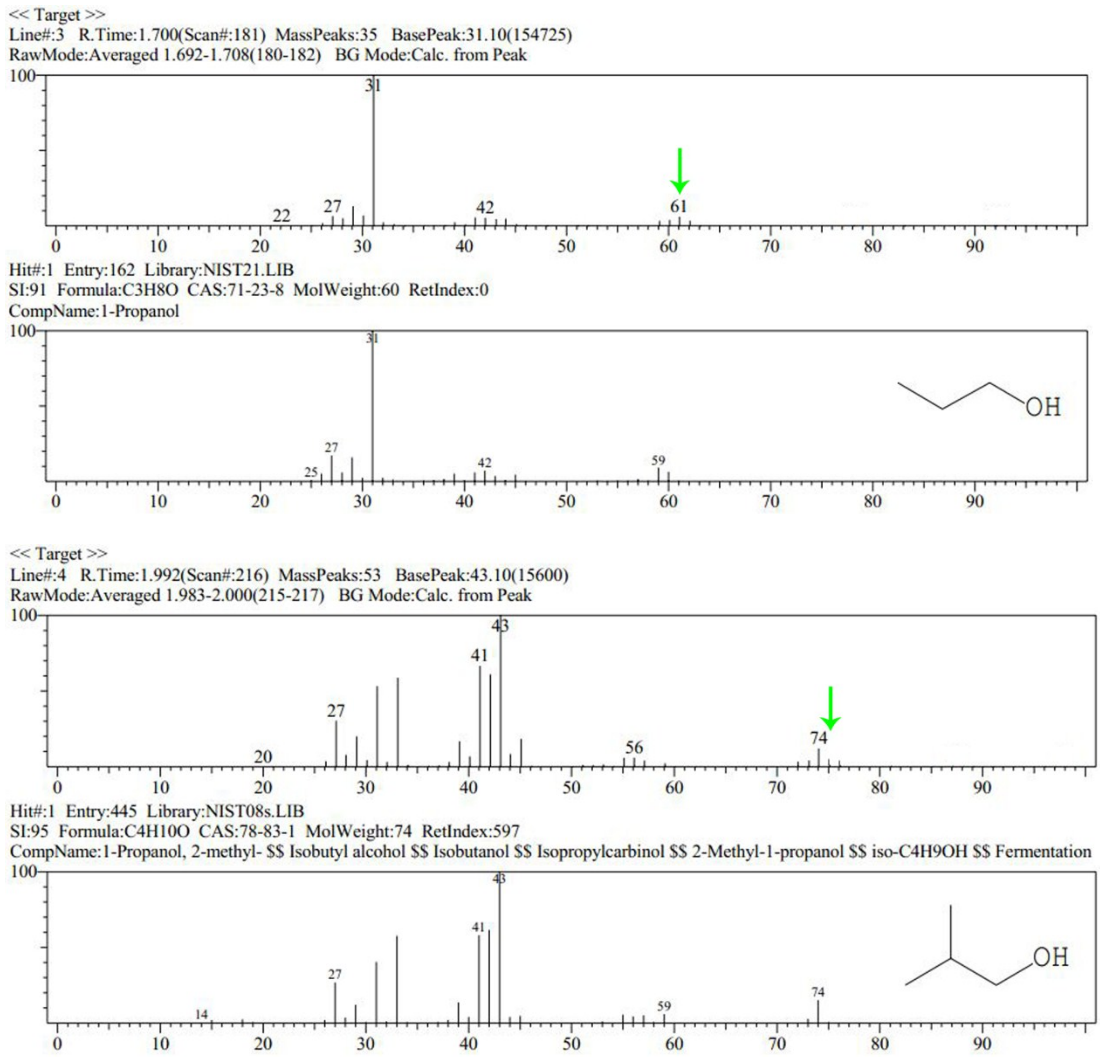

Fig. S4 GC-MS spectra of $^{13}C_2H_5OH$ tracer experiment. 25 μL $^{13}C_2H_5OH$ was used as tracer, other reaction conditions were the same as that of entry 1 in Table 1.

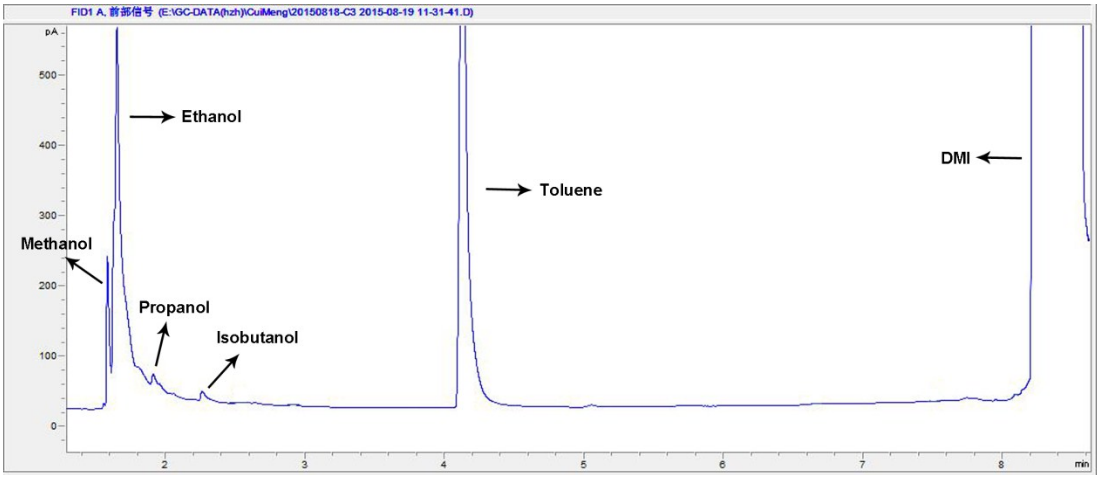

Fig. S5 Representative GC spectra of reaction solution after CO_2 hydrogenation with internal standard toluene. Reaction conditions were the same as that of entry 1 in Table 1.
